# Multimorbidity burden and patterns associated with DeepBrainNet‐derived brain–age gap in dementia‐free older adults: A community‐based study

**DOI:** 10.1002/alz.71647

**Published:** 2026-07-01

**Authors:** Xinyu Liu, Ming Mao, Cuicui Liu, Dige Ai, Jiacheng Wang, Tianyu Yu, Xiaodong Han, Yifei Ren, Xiaolei Han, Yi Dong, Lin Song, Shi Tang, Na Tian, Lin Cong, Kai Xu, Yifeng Du, Chengxuan Qiu, Yongxiang Wang

**Affiliations:** ^1^ Department of Neurology Shandong Provincial Hospital, Shandong University Jinan Shandong P.R. China; ^2^ Key Laboratory of Endocrine Glucose & Lipids Metabolism and Brain Aging Department of Neurology Ministry of Education Shandong Provincial Hospital affiliated to Shandong First Medical University Jinan Shandong P.R. China; ^3^ Shandong Institute of Brain Science and Brain‐Inspired Research, Medical Science and Technology Innovation Center Shandong First Medical University & Shandong Academy of Medical Science Jinan Shandong P.R. China; ^4^ State Key Laboratory of Cognitive Neuroscience and Learning Beijing Normal University Beijing P.R. China; ^5^ Innovation Center for Neurological Disorders and Department of Neurology National Clinical Research Center for Geriatric Diseases Xuanwu Hospital Capital Medical University Beijing China; ^6^ Aging Research Center, Department of Neurobiology, Care Sciences and Society Karolinska Institute‐Stockholm University Solna Sweden

**Keywords:** brain aging, cardiometabolic diseases, community‐based study, multimorbidity patterns

## Abstract

**INTRODUCTION:**

Emerging evidence has linked chronic diseases with structural brain measures; however, the relationship between multimorbidity patterns and brain–age gap is unclear.

**METHODS:**

This community‐based study involved 1151 dementia‐free older adults in Multimodal Interventions to Delay Dementia and Disability in Rural China (MIND‐China). Multimorbidity was defined as coexistence of two or more chronic diseases. Hierarchical cluster analysis was used to identify five patterns of multimorbidity. We additionally defined cardiometabolic multimorbidity as coexistence of two or more cardiometabolic diseases. The predicted brain age was estimated using DeepBrainNet. Data were analyzed using linear regression models.

**RESULTS:**

The number of chronic diseases, multimorbidity, and cardiometabolic multimorbidity were significantly associated with larger brain–age gap (*p* < 0.05). The multimorbidity clusters comprising cerebrovascular disease and metabolic disorders or biliary tract diseases, dorsopathies, anemia, and hearing problems were significantly correlated with larger brain–age gap (*p* < 0.05).

**DISCUSSION:**

The overall burden and cardiometabolic pattern of multimorbidity are associated with advanced brain aging in dementia‐free older adults.

## Highlights

- The presence and higher burden of multimorbidity were associated with a larger brain–age gap in older adults.
- Clusters of chronic diseases comprising metabolic or biliary tract disorders, dorsopathies, anemia, and hearing problems were associated with a larger brain–age gap.
- Cardiometabolic multimorbidity were associated with advanced brain age.

## 1 BACKGROUND

Multimorbidity, defined as the coexistence of two or more chronic health conditions in the same individual, is associated with reduced quality of life, impaired functional capacity, and substantial economic and societal burdens.^1^ Evidence has emerged that multimorbidity could contribute to accelerated brain aging.^2^, ^3^ However, prior research has focused primarily on the burden, rather than the patterns, of multimorbidity in relation to brain aging. Certain chronic health conditions often coexist in older people owing to shared pathophysiological mechanisms, which may exert synergistic effects on brain aging. By identifying groups of co‐coexisting chronic diseases, the data‐driven clustering analysis approach may reveal shared underlying mechanisms that are involved in accelerated brain aging. In addition to the data‐driven clustering approach, clinically defined multimorbidity patterns such as cardiometabolic multimorbidity may also contribute to accelerated brain aging through inflammatory pathways.^4^, ^5^ Exploring the relationship between multimorbidity patterns and structural brain aging is crucial for informing strategies aimed at preventing or delaying accelerated brain aging.

Brain aging can be assessed using neuroimaging markers such as global and regional brain volumes (e.g., frontal, temporal, and para‐hippocampal regions) as well as cerebral microvascular lesions (e.g., lacunes and white matter hyperintensities [WMH]).^3^, ^6^, ^7^, ^8^ In recent years, a novel approach has emerged to quantify brain aging using predicted brain age derived from neuroimaging features.^9^ The difference between predicted brain age and chronological age is termed the brain–age gap.^10^ A negative brain–age gap suggests a younger‐appearing brain relative to chronological age, whereas a positive gap indicates an older‐appearing brain and may reflect advanced brain aging. This approach allows for the direct assessment of accelerated or decelerated brain aging using structural brain magnetic resonance imaging (MRI) data. In particular, deep learning models (e.g., DeepBrainNet model) can effectively process large‐scale datasets and extract hierarchical structural brain features through layered neural network architectures.^10^ While previous studies have investigated the associations between multimorbidity and various structural brain MRI measures^3^, few have specifically examined multimorbidity patterns in relation to the brain–age gap.

Therefore, in this community‐based study, we first applied the DeepBrainNet model to estimate the predicted brain age and then, examined the associations of multimorbidity burden and patterns with the brain–age gap among dementia‐free older adults in rural China. We hypothesized that a higher burden and certain patterns of multimorbidity would be associated with a larger brain–age gap (i.e., a measure of accelerated brain aging) among dementia‐free older adults.

## 2 METHODS

### 2.1 Study design and participants

This community‐based cross‐sectional study used data from the Multimodal Interventions to Delay Dementia and Disability in Rural China (MIND‐China) project, one of the participating projects in the World‐Wide Finnish Geriatric Intervention Study to Prevent Cognitive Impairment and Disability Network.^11^ In March–September 2018, the baseline examinations were carried out during which a total number of 5765 residents who were aged ≥ 60 years and living in rural communities (52 villages) of Yanlou town, Yanggu county, western Shandong province were examined for MIND‐China, as previously reported.^12^, ^13^ Of these, a subsample of 1304 participants from 26 villages that were selected from all the 52 villages in Yanlou Town using cluster (village) ‐randomized sampling approach participated in the brain MRI sub‐study.^12^, ^14^ Of these, 153 participants were excluded due to invalid brain MRI data necessary for brain age prediction (i.e., missing T1 image, intracranial pathologies, or suboptimal image quality; *n* = 76), a diagnosis of prevalent dementia (*n* = 25), and missing data on diagnosis of chronic diseases (*n* = 52), leaving a final sample of 1151 subjects for the current analysis (Figure S1).

The Ethics Committee at Shandong Provincial Hospital approved the protocol of MIND‐China. Written informed consent was obtained from all participants or from their informants if they have severe cognitive impairment. Research within MIND‐China has been conducted in accordance with the ethical principles for medical research involving human subjects expressed in the Declaration of Helsinki. MIND‐China as an interventional study was registered in the Chinese Clinical Trial Registry (registration no.: ChiCTR1800017758).

### 2.2 Data collection and assessments

In March–September 2018, the trained staff collected data through face‐to‐face interviews, clinical examinations, cognitive assessments, and laboratory tests following a structured questionnaire, as previously reported.^15^ Education, alcohol intake, and smoking status were categorized as previously described.^12^ The definitions and identifications of chronic health conditions in this study were based on an operational framework that has been used in the Swedish National study on Aging and Care in Kungsholmen.^16^, ^17^, ^18^ In brief, a chronic disease was referred to as a disease that had a prolonged duration and either resulted in residual disability or deterioration in quality of life, or required long‐term care, treatment, or rehabilitation. All chronic diseases were classified and coded according to the International Classification of Diseases, 10th Revision (ICD‐10). In the present study, chronic health conditions were identified and defined using multiple data sources, including self‐reported physician diagnosis of a medical condition, physical and neurological examinations (e.g., blood pressure, weight, and height), laboratory tests (e.g., blood glucose and lipids), validated questionnaires (e.g., the Pittsburgh Sleep Quality Index, the 5‐item Geriatric Depression Scale, and the Mini‐Mental State Examination), and instrumental examinations (e.g., electrocardiogram). A total of 37 chronic diseases were assessed and defined. Chronic conditions with a prevalence of less than 5% were excluded as suggested in previous studies^19^, ^20^, which resulted in 19 chronic conditions that were included in the final analysis.

**RESEARCH IN CONTEXT**

1. **Systematic review**: We searched PubMed for literature examining the associations of multimorbidity parameters with structural brain measures and brain aging markers. Emerging evidence has linked multimorbidity with individual markers of structural brain aging. However, the association between multimorbidity patterns and predicted brain–age gap, an integrative marker of brain aging derived from neuroimaging‐based models, has yet to be investigated in the general population, especially in rural Chinese older adults.
2. **Interpretations**: This community‐based cross‐sectional study of rural Chinese older adults suggested that the presence, the increased burden, and the metabolic cluster of multimorbidity were associated with a large brain–age gap. In addition, cardiometabolic multimorbidity was associated with an advanced brain–age gap. These results suggest that multimorbidity, particularly cardiometabolic multimorbidity, may accelerate the brain aging process.
3. **Future directions**: Future prospective cohort studies should investigate the longitudinal association of multimorbidity with accelerated brain aging, as well as the underlying neuropathological mechanisms. Such research is crucial for informing the development of targeted interventions to delay brain aging and preserve cognitive health in older adults.

### 2.3 Assessment of multimorbidity burden and patterns

Multimorbidity was defined as the concurrent presence of two or more chronic health conditions.^16^ Multimorbidity burden was defined as the total number of chronic conditions present in an individual. Multimorbidity patterns were identified using hierarchical clustering, as previously described.^13^, ^21^ In brief, we quantified pairwise similarities between diseases using Pearson's correlation coefficients, which were then converted to a distance metric defined as $\mathrm{Distanc}e_{ij}=1-r_{ij}$, where $r_{ij}$ denotes the correlation between disease i and disease j.^22^ Hierarchical clustering was performed using Ward's minimum variance method, which iteratively merges clusters to minimize the increase in within‐cluster variance. Based on previous literature and considerations of clinical interpretability, we ultimately identified five multimorbidity clusters, with each cluster represented by its centroid defined using the maximum within‐cluster distance.^13^, ^23^

**FIGURE 1:**
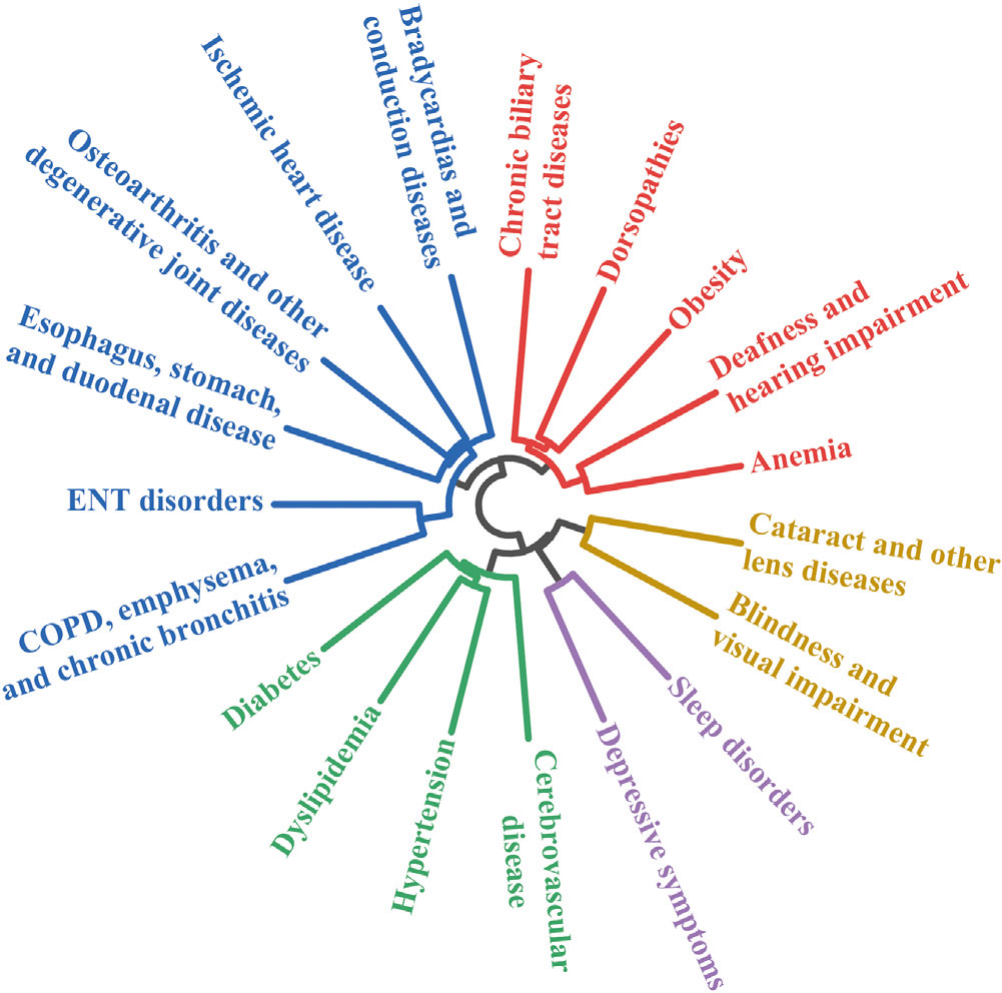
Dendrogram of multimorbidity clusters (*n* = 1151). Multimorbidity cluster 1 included cerebrovascular disease, hypertension, dyslipidemia, and diabetes; cluster 2 included sleep disorders and depressive symptoms; cluster 3 included blindness, visual impairment, and cataract and other lens diseases; cluster 4 included anemia, chronic biliary tract diseases, dorsopathies, obesity, and deafness or hearing impairment; and cluster 5 included osteoarthritis and other degenerative joint diseases, ischemic heart disease, ENT disorders, COPD, emphysema, chronic bronchitis, esophagus, stomach, and duodenal disease, and bradycardias and conduction diseases. ENT, ear, nose, and throat; COPD, chronic obstructive pulmonary disease

Figure 1 illustrates five multimorbidity clusters derived from 19 chronic diseases. Cluster 1 included cerebrovascular disease, hypertension, dyslipidemia, and diabetes; cluster 2 consisted of sleep disorders and depressive symptoms; cluster 3 encompassed blindness, visual impairment, and cataract and other lens diseases; cluster 4 included anemia, chronic biliary tract diseases, dorsopathies, obesity, and deafness or hearing impairment; and cluster 5 included osteoarthritis and other degenerative joint diseases, ischemic heart disease, ear, nose, and throat disorders, chronic obstructive pulmonary disease (COPD), emphysema, chronic bronchitis, esophagus, stomach, and duodenal disease, and bradycardias and conduction diseases.

### 2.4 Assessment of cardiometabolic diseases and cardiometabolic multimorbidity

Cardiometabolic diseases (CMD) included heart disease, diabetes, and cerebrovascular disease, and cardiometabolic multimorbidity (CMM) was defined as the coexistence of at least two of these three conditions, as proposed in previous studies.^24^, ^25^, ^26^ Heart disease comprised ischemic heart disease, atrial fibrillation, and heart failure ascertained through self‐reported physician diagnosis of the respective conditions or electrocardiogram examination. Diabetes was defined according to self‐reported physician diagnosis of diabetes or fasting blood glucose ≥ 7.0 mmol/L or current use of glucose‐lowering medication. Cerebrovascular disease was identified through self‐reported physician diagnosis of cerebrovascular disease.^27^

### 2.5 Brain MRI data acquisition and processing

Structural brain MRI scans were performed on either the Philips Ingenia 3.0T MR scanner in Shandong Southwestern Lu Hospital (*n* = 1178) or the Philips Archiva 3.0T MR scanner in Liaocheng Hospital (*n* = 136). The core MRI sequences and parameters for different sequences were fully reported elsewhere.^12^, ^14^

We used the DeepBrainNet model to estimate predicted brain age from T1‐weighted images. The model was trained using 11729 MRI scans from a highly diverse cohort of individuals aged 3–95 years, drawn from 16 imaging databases across different studies and geographic locations.^10^ Raw T1‐weighted images were skull‐stripped using Advanced Normalization Tools (ANTs) and nonlinearly registered to the MNI152 a2009c template using sMRIprep before being processed by the DeepBrainNet model. The brain–age gap was initially calculated as the difference between predicted brain age and chronological age. Model performance was assessed using the mean absolute error (MAE). The DeepBrainNet model yielded a MAE of 12.73 years for brain age prediction (Figure S2). In addition, brain–age gap was significantly correlated with chronological age (r = ‐0.072, p = 0.015). This suggests the presence of regression to the mean, whereby predicted brain age tends to be overestimated in younger individuals and underestimated in older individuals, resulting in an age‐dependent bias in the brain–age gap.^28^ We used a linear bias‐correction approach to address this issue.^28^, ^29^ After the correction, the MAE was reduced to 5.27 years, and the correlation between chronological age and brain–age gap was no longer significant (*r* = 0.003, *p* = 0.913).

### 2.6 Statistical analysis

Characteristics of the study participants were described as frequencies (%) for categorical variables and mean (standard deviation, SD) for continuous variables. Differences in demographics, lifestyles, and prevalence of chronic conditions and multimorbidity by sex were compared using chi‐square test for categorical variables and Wilcoxon test for skewed continuous variables.

We first examined the associations of individual chronic diseases and the burden of chronic diseases with brain–age gap. Then, we used general linear regression models to investigate the associations of multimorbidity patterns with brain–age gap. Model 1 was adjusted for chronological age, sex, education, and MRI scan centers; and model 2 was additionally adjusted for smoking status and alcohol intake.

Statistical analyses were performed using Origin 2022 (Origin 2022 Feature Highlights at originlab.com; http://originlab.com/2022) and R 3.6.3 for Windows (R Foundation for Statistical Computing, Vienna, Austria. http://www.R‐project.org/). Two‐tailed *p *< 0.05 was considered statistically significant.

## 3 RESULTS

### 3.1 Characteristics of the study participants

Of the 1151 participants, the mean age was 70.8 (SD, 4.2) years, 58.5% were females, and 33.9% had no formal schooling. Compared with males, females had a lower predicted brain age and were more likely to have dyslipidemia, diabetes, sleep disorders, anemia, depressive symptoms, ischemic heart disease, and chronic biliary tract diseases, but less likely to have COPD, emphysema, chronic bronchitis, bradycardias, conduction disease, and deafness and hearing impairment (all *p *< 0.05) (Table 1).

**TABLE 1:** Characteristics of study participants in the total sample and by sex (*n* = 1151)

Characteristics	Total sample (*n* = 1151)	Men(*n* = 478)	Women (*n* = 673)	*p*‐value
**Chronological age, years**	70.8 (4.2)	70.9 (4.3)	70.7 (4.2)	0.529
**Education, *n* (%)**				<0.001
No formal schooling	390 (33.9)	41 (8.6)	349 (51.9)	
Primary school	525 (45.6)	241 (50.4)	284 (42.2)	
Middle school or above	236 (20.5)	196 (41.0)	40 (5.9)	
**Predicted brain age, years**	58.3 (7.6)	59.4 (7.2)	57.6 (7.9)	<0.001
**Predicted brain–age gap, years**	−0.1 (6.6)	0.8 (6.4)	−0.8 (6.7)	<0.001
**Alcohol intake,** ^a^ ***n* (%)**				<0.001
Never	702 (61.0)	82 (17.2)	620 (92.1)	
Former	80 (7.0)	77 (16.1)	3 (0.4)	
Current	361 (31.4)	312 (65.3)	49 (7.3)	
**Smoking status, *n* (%)**				<0.001
Never	788 (68.5)	123 (25.7)	665 (98.8)	
Former	132 (11.5)	132 (27.6)	0 (0)	
Current	231 (20.1)	223 (46.7)	8 (1.2)	
**Clinical conditions, *n* (%)**				
Hypertension	786 (68.3)	325 (68.0)	461 (68.5)	0.906
Dyslipidemia	285 (24.8)	79 (16.5)	206 (30.6)	<0.001
Diabetes	171 (14.9)	55 (11.5)	116 (17.2)	0.009
Cataract and other lens diseases	116 (10.1)	51 (10.7)	65 (9.7)	0.644
Blindness or visual impairment	95 (8.3)	37 (7.7)	58 (8.6)	0.671
Sleep disorders	437 (38.0)	160 (33.5)	277 (41.2)	0.010
Depressive symptoms	81 (7.0)	22 (4.6)	59 (8.8)	0.009
Chronic biliary tract diseases	71 (6.2)	17 (3.6)	54 (8.0)	0.003
Osteoarthritis and other degenerative joint diseases	204 (17.7)	78 (16.3)	126 (18.7)	0.330
Ischemic heart disease	233 (20.2)	75 (15.7)	158 (23.5)	0.002
ENT disorders	129 (11.2)	64 (13.4)	65 (9.7)	0.060
COPD, emphysema, and chronic bronchitis	66 (5.7)	38 (7.9)	28 (4.2)	0.009
Esophagus, stomach, and duodenum diseases	103 (8.9)	51 (10.7)	52 (7.7)	0.106
Bradycardias and conduction diseases	77 (6.7)	49 (10.3)	28 (4.2)	<0.001
Dorsopathies	70 (6.1)	24 (5.0)	46 (6.8)	0.253
Obesity	96 (8.3)	32 (6.7)	64 (9.5)	0.111
Deafness and hearing impairment	226 (19.6)	115 (24.1)	111 (16.5)	0.002
Anemia	184 (16.0)	57 (11.9)	127 (18.9)	0.002
Cerebrovascular disease	146 (12.7)	68 (14.2)	78 (11.6)	0.217
Multimorbidity,^b^ *n* (%)	958 (83.2)	387 (81.0)	571 (84.8)	0.098
**Clusters of multimorbidity,** ^c^ ***n* (%)**				
Cluster 1	900 (78.2)	362 (75.7)	538 (79.9)	0.103
Cluster 2	464 (40.3)	170 (35.6)	294 (43.7)	0.007
Cluster 3	192 (16.7)	81 (16.9)	111 (16.5)	0.902
Cluster 4	507 (44.0)	195 (40.8)	312 (46.4)	0.070
Cluster 5	569 (49.4)	245 (51.3)	324 (48.1)	0.327

*Note*: Data were mean (standard deviation), unless otherwise specified.

Abbreviations: COPD, chronic obstructive pulmonary disease; ENT, ear, nose, and throat.

^a^ The number of participants with missing values was eight for alcohol intake, including seven men and one woman.

^b^ Multimorbidity was defined as coexistence of two or more of the 19 chronic diseases.

^c^Multimorbidity cluster 1 included cerebrovascular disease, hypertension, dyslipidemia, and diabetes; cluster 2 included sleep disorders and depressive symptoms; cluster 3 included blindness, visual impairment, and cataract and other lens diseases; cluster 4 included anemia, chronic biliary tract diseases, dorsopathies, obesity, and deafness or hearing impairment; and cluster 5 included osteoarthritis and other degenerative joint diseases, ischemic heart disease, ENT disorders, COPD, emphysema, chronic bronchitis, esophagus, stomach, and duodenal disease, and bradycardias and conduction diseases.

### 3.2 Associations of individual chronic diseases with brain–age gap

Of the 19 chronic diseases, controlling for demographic and lifestyle factors, a significant association with a larger brain–age gap was observed for hypertension (β = 1.45; 95% confidence interval [CI]: 0.74, 2.16), diabetes (β = 1.62; 95% CI: 0.69, 2.55), obesity (β = 1.96; 95% CI: 0.78, 3.15), cerebrovascular disease (β = 1.52; 95% CI: 0.53, 2.51), and blindness or visual impairment (β = 1.76; 95% CI: 0.56, 2.95) (Table 2). Besides, while deafness and hearing impairment were marginally associated with a larger brain–age gap, none of the other chronic diseases examined showed a significant association with the brain–age gap (Table 2).

**TABLE 2:** Association of individual chronic diseases with brain–age gap (*n* = 1151)

	**β Coefficient (95% confidence interval), brain–age gap (years)**			
**Chronic diseases**	**Model 1** ^a^	***p‐*Value**	**Model 2** ^a^	***p*‐value**
Hypertension	**1.41 (0.70, 2.11)**	**<0.001**	**1.45 (0.74, 2.16)**	**<0.001**
Dyslipidemia	0.21 (−0.56, 0.98)	0.597	0.19 (−0.58, 0.97)	0.626
Diabetes	**1.63 (0.71, 2.56)**	**<0.001**	**1.62 (0.69, 2.55)**	**<0.001**
Cataract and other lens diseases	−0.37 (−1.46, 0.73)	0.509	−0.42 (−1.52, 0.68)	0.454
Blindness or visual impairment	**1.74 (0.54, 2.94)**	**0.004**	**1.76 (0.56, 2.95)**	**0.004**
Sleep disorders	0.54 (−0.14, 1.22)	0.119	0.55 (−0.14, 1.23)	0.116
Depression	0.33 (−0.97, 1.62)	0.623	0.32 (−0.98, 1.62)	0.631
Chronic biliary tract and gallbladder diseases	−0.03 (−1.40, 1.35)	0.967	−0.02 (−1.40, 1.35)	0.973
Osteoarthritis and other degenerative joint diseases	−0.02 (−0.88, 0.84)	0.962	−0.00 (−0.87, 0.86)	0.995
Ischemic heart disease	0.62 (−0.21, 1.44)	0.144	0.58 (−0.25, 1.42)	0.170
ENT disorders	0.03 (−1.01, 1.08)	0.950	0.02 (−1.03, 1.07)	0.971
COPD, emphysema, and chronic bronchitis	1.34 (−0.08, 2.76)	0.064	1.22 (−0.22, 2.66)	0.098
Esophagus, stomach, and duodenum diseases	−0.65 (−1.81, 0.50)	0.266	−0.66 (−1.81, 0.50)	0.265
Bradycardias and conduction diseases	0.05 (−1.27, 1.38)	0.938	0.09 (−1.24, 1.41)	0.899
Dorsopathies	0.60 (−0.78, 1.97)	0.395	0.64 (−0.74, 2.02)	0.362
Obesity	**1.97 (0.79, 3.16)**	**0.001**	**1.96 (0.78, 3.15)**	**0.001**
Deafness and hearing impairment	0.84 (−0.01, 1.70)	0.054	0.81 (−0.05, 1.67)	0.066
Anemia	0.76 (−0.15, 1.67)	0.101	0.73 (−0.18, 1.64)	0.114
Cerebrovascular disease	**1.54 (0.55, 2.53)**	**0.002**	**1.52 (0.53, 2.51)**	**0.003**

*Note*: Bold formatting used to highlight statistically significant results.

Abbreviations: COPD, chronic obstructive pulmonary disease; ENT, ear, nose, and throat; MRI magnetic resonance imaging.

^a^ Model 1 was adjusted for chronological age, sex, education, and MRI scan centers; and Model 2 was further adjusted for smoking status and alcohol intake.

### 3.3 Association of multimorbidity burden with brain–age gap

The number of chronic diseases, as a continuous variable, was significantly associated with the brain–age gap (multivariable‐adjusted β‐coefficient = 0.53; 95% CI: 0.34, 0.73) (Table 3). The presence of multimorbidity was associated with multivariable‐adjusted β‐coefficient of 1.57 (95% CI: 0.68, 2.45) for brain–age gap (Table 3). In addition, an increased number of CMD was associated with a larger brain–age gap, with the multivariable‐adjusted β coefficient being 1.11 (95% CI: 0.61, 1.61) (Table 3). The presence of CMM was associated with the multivariable‐adjusted β coefficient of 2.47 (95% CI: 1.27, 3.68).

**TABLE 3:** Associations of the presence and burden of multimorbidity with brain–age gap (*n* = 1151)

Multimorbidity measures	No. of participants	β Coefficient (95% confidence interval), brain–age gap (years)	
		Model 1^a^	Model 2^a^
**Chronic diseases**			
No. of chronic diseases (range 0–9)	1151	**0.54 (0.34, 0.73)** ^*^	**0.53 (0.34, 0.73)** ^*^
Presence of multimorbidity			
No	193	0.00 (reference)	0.00 (reference)
Yes	958	**1.60 (0.72, 2.48)** ^*^	**1.57 (0.68, 2.45)** ^*^
**CMDs**			
No. of CMDs (range 0–3)	1151	**1.12 (0.62, 1.62)** ^*^	**1.11 (0.61, 1.61)** ^*^
Presence of CMM			
No	1057	0.00 (reference)	0.00 (reference)
Yes	94	**2.49 (1.29, 3.69)** ^*^	**2.47 (1.27, 3.68)** ^*^

*Note*: Bold formatting used to highlight statistically significant results.

Abbreviations: CMD, cardiometabolic disease; CMM, cardiometabolic multimorbidity; MRI, magnetic resonance imaging.

^a^ Model 1 was adjusted for chronological age, sex, education, and MRI scan centers; and Model 2 was further adjusted for smoking status and alcohol intake.

* *p *< 0.001.

### 3.4 Association of multimorbidity patterns with brain–age gap

Among the five multimorbidity clusters, cluster 1, comprising cerebrovascular disease, hypertension, dyslipidemia, and diabetes, and cluster 4, comprising chronic biliary tract diseases, dorsopathies, obesity, anemia, and deafness and hearing impairment, were significantly associated with a larger brain–age gap, with β coefficients being 1.97 (95% CI: 1.17, 2.77) and 1.25 (95% CI: 0.58, 1.92), respectively. The other three multimorbidity clusters were not significantly associated with the brain–age gap (Table 4).

**TABLE 4:** Associations of multimorbidity patterns with brain–age gap (*n* = 1151)

Clusters of multimorbidity^a^	No. of participants	β Coefficient (95% confidence interval), brain–age gap (years)	
		Model 1^b^	Model 2^b^
Cluster 1	900	**1.95 (1.16, 2.75)** ^*^	**1.97 (1.17, 2.77)** ^*^
Cluster 2	464	0.46 (−0.22, 1.13)	0.46 (−0.22, 1.14)
Cluster 3	192	0.72 (−0.16, 1.61)	0.71 (−0.18, 1.60)
Cluster 4	507	**1.27 (0.60, 1.94)** ^*^	**1.25 (0.58, 1.92)** ^*^
Cluster 5	569	0.39 (−0.27, 1.05)	0.37 (−0.30, 1.03)

Abbreviation: MRI, magnetic resonance imaging.

^a^Multimorbidity cluster 1 included cerebrovascular disease, hypertension, dyslipidemia, and diabetes; cluster 2 included sleep disorders and depressive symptoms; cluster 3 included blindness, visual impairment, and cataract and other lens diseases; cluster 4 included anemia, chronic biliary tract diseases, dorsopathies, obesity, and deafness or hearing impairment; and cluster 5 included osteoarthritis and other degenerative joint diseases, ischemic heart disease, ear, nose, and throat disorders, chronic obstructive pulmonary disease, emphysema, chronic bronchitis, esophagus, stomach, and duodenal disease, and bradycardias and conduction diseases.

^b^Model 1 was adjusted for chronological age, sex, education, and MRI scan centers; and Model 2 was further adjusted for smoking status and alcohol intake.

^*^ *p *< 0.001.

## 4 DISCUSSION

The main findings of the community‐based study of older adults in rural China were summarized as follows: (1) the presence and an increased burden of multimorbidity were associated with a larger brain–age gap; (2) the presence of CMM and the number of CMDs were correlated with advanced brain age; and (3) multimorbidity clusters comprising cerebrovascular disease and metabolic disorders or biliary tract diseases, dorsopathies, anemia, and hearing problems were associated with a larger brain–age gap. To the best of our knowledge, this is the first study to quantify the associations of multimorbidity burden and patterns with brain–age gap in a general population of older adults in rural China.

Previous studies have linked chronic diseases with impaired structural brain measures and brain activity. For instance, a study from the UK Biobank that involved 57 chronic diseases found that an increased number of chronic diseases was associated with larger WMH volume and smaller volumes of the hippocampus, gray matter, and total brain tissue.^3^ Similarly, the Baltimore Longitudinal Study of Aging (BLSA) showed that a higher burden of multimorbidity was associated with relatively lower activity in the frontal and temporal lobes, brain regions critically involved in memory, planning, decision‐making, and emotion.^30^ However, the Midlife in the United States Refresher Neuroscience Project (*n* = 138, aged 25–74 years) found no evidence for the association of multimorbidity with the brain–age gap assessed using the brainageR model.^31^ Differences in demographic characteristics of the study samples as well as variations in models of quantifying brain age may partly explain the inconsistent findings across studies. Our study found that, on average, the brain–age gap increased by 0.53 years for each additional chronic disease in older adults. Importantly, the brain–age gap represents a comprehensive quantitative metric of brain aging, capturing information beyond what individual structural brain imaging markers alone can provide.

Previous studies have linked individual chronic metabolic health conditions with an increased brain age.^3^, ^4^, ^32^ In a cross‐sectional study of cognitively unimpaired populations from Korea and the United Kingdom, diabetes was associated with an approximately 1‐ to 2‐year increase in brain age, whereas hypertension was linked to a 0.5‐ to 0.8‐year increase in brain age.^4^ Similarly, our study found that hypertension was associated with 1.45 years of increased brain age, and the corresponding figure was 1.52 years for cerebrovascular disease, 1.62 years for diabetes, and 1.96 years for obesity. Taken together, these findings reinforce a robust and consistent association between metabolic‐related chronic conditions and accelerated brain aging. Furthermore, vision loss or hearing impairment was associated with a larger brain–age gap, suggesting that impairments in sensory system are associated with advanced brain aging. This is in line with current evidence showing that sensory deficits (e.g., hearing or vision impairment) are associated with an increased risk of Alzheimer's disease and dementia.^33^ In addition, a case–control study suggested that major depressive disorder was associated with a higher brain–age gap among individuals aged 12–82 years,^34^ whereas our community‐based study of older adults showed insufficient evidence for the association of depressive symptoms with an increased brain–age gap. Variations in the study design, demographic characteristics of study participants, and defining methods of depressive conditions may partly contribute to different findings across studies. Taken together, our community‐based study extends previous findings by providing evidence for the associations between a broad range of chronic health conditions (e.g., metabolic and sensory disorders) and an increased brain–age gap among rural‐dwelling Chinese older adults.

Previous studies have shown a clear association between individual cardiometabolic conditions and advanced brain aging.^4^ However, few studies have examined the associations of multimorbidity patterns with brain aging markers. A study from UK Biobank reported that the metabolic syndrome, defined based on obesity, hypertriglyceridemia, low high‐density lipoprotein levels, hypertension, and hyperglycemia, was associated with an increased brain–age gap.^35^ The mean brain–age gap increased from ‐0.37 years among people without any of the five metabolic syndrome components to 0.91 years among those with all the five components, corresponding to a 1.28‐year difference in brain age. We explored the relationship between multimorbidity patterns and brain–age gap, in which multimorbidity patterns were defined from both clinical and statistical perspectives. Using the clinical approach, we defined CMM based on three common CMDs (i.e., heart disease, cerebrovascular disease, and diabetes). Our study suggested that the presence of CMM was associated with 2.47 years of brain–age gap. Interestingly, the data‐driven hierarchical clustering approach identified a multimorbidity cluster comprising cerebrovascular disease, hypertension, dyslipidemia, and diabetes, which was associated with a 1.97‐year increase in brain–age gap. Thus, our study provides evidence that cardiometabolic comorbidity patterns, defined and identified using both clinical‐based and data‐driven approaches, are consistently associated with advanced brain aging. In addition, population‐based studies of middle‐aged and older adults have linked CMM patterns with all‐cause dementia, Alzheimer's disease, and vascular dementia.^26^, ^36^ Collectively, these findings suggest that CMM patterns are associated with advanced brain aging and dementia outcomes, underscoring its potential as a target for preventive and therapeutic interventions to promote brain health and reduce dementia risk among middle‐aged and older adults. Therefore, future studies are imperative to clarify whether comprehensive management of multiple cardiometabolic conditions may slow brain aging and delay onset of dementia disorders.

Inflammatory processes may underlie the association between metabolic multimorbidity and accelerated brain aging. Chronic excess of free fatty acids and glucose may activate inflammatory pathways through direct or indirect induction of reactive oxygen species.^37^ Furthermore, chronic inflammation and oxidative stress have been involved in neurodegeneration, amyloid‐β and tau deposition, and cerebrovascular dysfunction, thereby linking metabolic multimorbidity to advanced brain aging and cognitive disorders.^38^ Future studies should further elucidate biological mechanisms underlying the associations between CMM, brain aging, and cognitive phenotypes, and to explore whether optimal management of these metabolic conditions could mitigate brain aging and neurodegenerative processes.

Our analysis also revealed that the multimorbidity pattern comprising anemia, biliary tract diseases, obesity, dorsopathies, and hearing problems was associated with a larger brain–age gap. This association is likely driven by the cumulative impact of these chronic health conditions via multiple mechanisms. Specifically, anemia may compromise cerebral oxygen delivery^39^; obesity‐related oxidative stress and inflammation can impair mitochondrial function and diminish neuronal repair capacities^40^; and chronic hearing impairment may affect sensory input, impose an excessive cognitive load, and accelerate cortical atrophy.^41^, ^42^ Notably, the data‐driven hierarchical clustering analysis identified distinct groups of coexisting chronic conditions beyond predefined categories, offering a more comprehensive characterization of multimorbidity complexity in older adults.

Our study engaged a large sample of community‐dwelling older adults in rural China, a sociodemographic group that is disproportionately affected by dementia disorders, yet remains substantially underrepresented in research on brain aging and dementia.^43^ Thus, findings from our study could partly address the important knowledge gap. In addition, our study was the first to quantify the association of multimorbidity patterns with brain–age gap, in which multimorbidity patterns were defined from both clinical (i.e., CMM) and statistical perspectives (i.e., using the data‐driven hierarchical clustering approach to identify multimorbidity clusters). However, our study has several limitations. First, because of the cross‐sectional nature of our study design, we cannot infer causality for any of the observed associations and the cross‐sectional associations may be affected by selective survival bias that often leads to underestimation of the true associations. Future prospective cohort studies are needed to determine the direction and magnitude of these observed associations. In addition, participants in the brain MRI sub‐study were relatively younger, healthier, and more educated than those in the MIND‐China total sample,^12^ which could also weaken the observed associations between multimorbidity measures and brain–age gap. Finally, our study participants were recruited from a single rural area in western Shandong province. Due to substantial differences in sociodemographic characteristics, levels of economic development, and well‐being across China (e.g., rural vs. urban areas and eastern vs. western regions), caution is warranted when generalizing our research findings to different populations in China, including rural populations.

In summary, our community‐based study found that the presence, an increased burden, and certain patterns (e.g., CMM) of multimorbidity were associated with advanced brain aging that warrants particular attention in future research. These findings have potential implications for understanding brain aging and underlying mechanisms. Given that MIND‐China is an ongoing multimodal interventional study that focuses on cognitive outcomes and brain health, future research is warranted to investigate whether multidomain preventive and therapeutic interventions could reduce the burden of multimorbidity, and CMM in particular, thus contributing to healthy brain aging and cognitive health as well.

## CONFLICT OF INTEREST STATEMENT

The authors declare no conflicts of interest. Author disclosures are available in the Supporting Information.

## CONSENT STATEMENT

Written informed consent was obtained from all participants, or in the case of dementias persons, from an informant (usually a guardian or a family member).

## Supporting information



Supporting material: alz71647‐Sup‐0002‐ICMJE.pdf

Supporting material: alz71647‐Supp‐0001‐SuppMatt.docx
