# Supplementary material for: Multimorbidity burden and patterns associated with DeepBrainNet‐derived brain–age gap in dementia‐free older adults: A community‐based study
Source: Alzheimers Dement. 2026 Jul 1;22(7):e71647. doi: 10.1002/alz.71647 (PMC13322993; doi:10.1002/alz.71647)
Supplement: Supplementary file 2 — Supporting material: alz71647‐Supp‐0001‐SuppMatt.docx [file ALZ-22-e71647-s002.docx]

**SUPPLEMENTARY MATERIAL**

**Multimorbidity burden and patterns associated with DeepBrainNet-derived brain age gap in dementia-free older adults: a community-based study**

Liu X**^†^**, Mao M**^†^**, et al.

**^†^**These authors contributed equally to this work.

**CONTENTS**

**Supplementary Figures**

1. **Supplementary Figure 1.** Flowchart of the study participants.
2. **Supplementary Figure 2.** Scatterplots on correlations of chronological age with predicted brain age, brain age gap, and adjusted brain age gap (n=1151).

MIND-China brain MRI sub-study (August 2018-December 2020), n=1304

76 were excluded due to:

- missing T1 image (n=12);
- intracranial pathologies (n=12);
- suboptimal image quality (n=52).

Subjects with optimal brain MRI image quality (analytical sample involving brain structure for predicted brain age), n=1228

77 were excluded due to:

- incomplete data on multimorbidity (n=52);
- prevalent dementia (n=25).

Analytical sample of dementia-free participants with multimorbidity data, n=1151

**Supplementary Figure 1. Flowchart of the study participants**

**Abbreviation:** MIND-China, Multimodal Interventions to Delay Dementia and Disability in Rural China; MRI, magnetic resonance imaging.


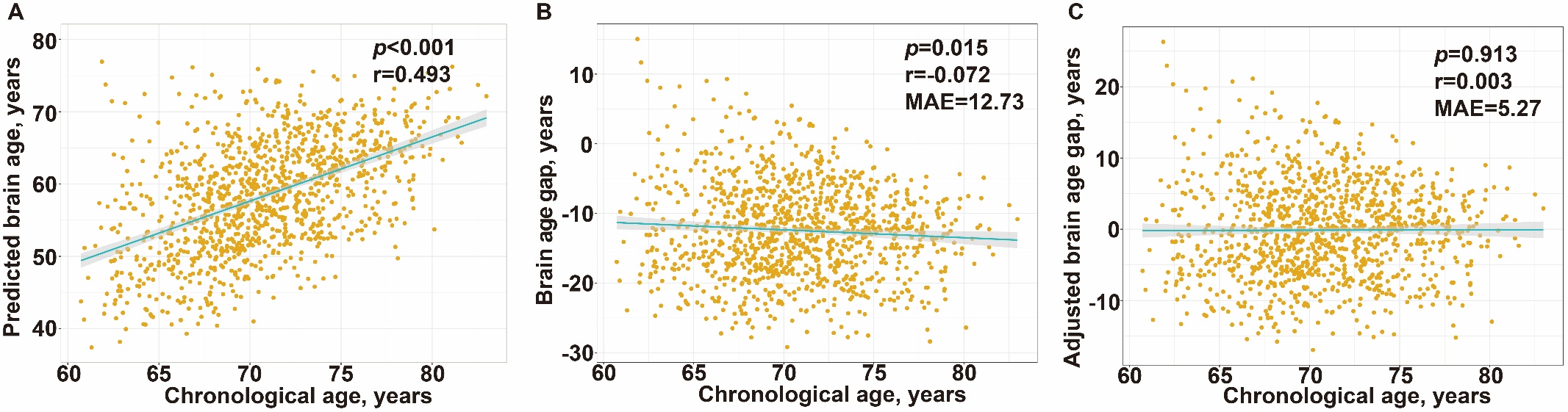


**Supplementary Figure 2. Scatterplots on correlations of chronological age with (A) predicted brain age, (B) brain age gap, and (C) adjusted brain age gap (n=1151)**

Note: The green line represents the best fit regression line and the shaded areas represent the 95% confidence intervals. The r value indicates Pearson’s correlation coefficient and p is for test of significance of r value.

**Abbreviations:** MAE, mean absolute error.
